# Clinical characteristics and risk factors for poor prognosis among HIV patients with Talaromyces marneffei bloodstream infection

**DOI:** 10.1186/s12879-021-06232-2

**Published:** 2021-06-01

**Authors:** Jianjun Sun, Weiwei Sun, Yang Tang, Renfang Zhang, Li Liu, Yinzhong Shen, Jiangrong Wang, Jun Chen, Tangkai Qi, Zhenyan Wang, Wei Song, Yixiao Lin, Shuibao Xu, Hongzhou Lu

**Affiliations:** 1 Department of Infection and Immunity, Shanghai Public Health Clinical Center, Shanghai, China; 2 Department of Obstetrics and Gynecology, Shanghai Public Health Clinical Center, Shanghai, China; 3 Department of Infectious Disease, Huashan Hospital Affiliated to Fudan University, Shanghai, China; 4 Department of Internal Medicine, Shanghai Medical College, Fudan University, Shanghai, China

**Keywords:** HIV infection, AIDS, Talaromyces marneffei, Bloodstream infection, Prevalence, Clinical characteristics, Poor prognosis, Risk factors

## Abstract

**Background:**

Talaromyces marneffei (TM) bloodstream infection is common in Acquired Immunodeficiency Syndrome (AIDS) patients with extreme immunodeficiency in Southeast Asia and South China, however, clinical case study on TM bloodstream infection is scarce. We retrospectively analyzed the clinical characteristics of TM bloodstream infection in hospitalized AIDS patients and determined the outcomes of hospitalization after diagnosis in our hospital over the past 5 years.

**Methods:**

From January 2015 to July 2020, 87 cases of TM detected by blood culture in patients admitted to our center were collected. The admission complaints, blood cells, biochemistry, CD4 and CD8 cell counts and 1,3-β-D-glucan (BDG), procalcitonin (PCT), CRP level on the day of blood culture test, and outcomes during hospitalization were analyzed. Logistic regression analysis was performed for the risk factors for poor prognosis (60 cases). Spearman correlation analysis was used to analyze the correlation between peripheral blood cells, albumin and the time required for TM turnaround in blood culture. The difference was statistically significant when the *P* value was < 0.05.

**Results:**

A total of 87 patients were collected, with a median age of 34 years, a median hemoglobin of 94 g/L and CD4 count of 7/μl. The rate of TM bloodstream infection among all in-hospital patients increased from 0.99% in 2015 to 2.09% in 2020(half year). Patients with TM bloodstream infection with CD8 count < 200/μl had a 12.6-fold higher risk of poor prognosis than those with CD8 count > 200/μl (*p* = 0.04), and those with BDG < 100 pg/mL had a 34.9-fold higher risk of poor prognosis than those with BDG > 100 pg/mL (*p* = 0.01).

**Conclusions:**

TM bloodstream infection is becoming more common in advanced AIDS patients in endemic areas. For those patients with extremely low CD4 and CD8 cell counts below 200/μl is with an increased risk of poor prognosis.

## Background

AIDS patients are susceptible to multiple opportunistic infections at the late stage, especially when the CD4 count is below 50/μl [1, 2]. The spectrum of bloodstream infection varies among people living with AIDS in different geographical settings [3]. *Talaromyces marneffei* (TM, formerly *Penicilliosis marneffei*) is endemic in Southeast Asia [4], Northeastern India [5], and Southern China [6]. The first natural human case of *Talaromycosis* (formerly *Penicillosis*) was reported in 1973 and involved an American minister with Hodgkin’s disease who lived in Southeast Asia [7]. TM was first reported in mainland China by Z.L. Deng in 1984 [8] and in Vietnam by T.V. Hien in 2001 [9]. Recently, TM has become an emerging pathogen in immunocompromised patients in mainland China [7]; furthermore, the number of confirmed cases of TM is increasing quickly in mainland China [10]. The incidence rates of TM infection range from 4 to 14%, with an associated mortality of 10–30% [11]. Reports of TM disease are common in the AIDS population in the late stage. A meta-analysis [12] showed that the prevalence of TM among HIV patients in China ranged from 0.2% (95% CI: 0.1–0.5%) to 26.5% (95% CI: 16.2–43.5%). South China had the highest prevalence, estimated at 15.0% (95% CI: 11.0–20.4%), while Southwest China had the lowest prevalence, estimated at 0.3% [13]. It is estimated that there will be 4951 TM cases per year in patients with AIDS in southern China by 2050, and the endemic areas are increasing [12]. The disease is mostly localized to the lungs and skin and is related to exposure by inhalation or direct contact TM [14, 15]. However, in clinical settings, because the onset of the disease is relatively insidious and mild, the clinical symptoms are non-specific, and patients often experience delayed diagnosis or misdiagnosis by doctors who are not familiar with the symptoms of TM disease. Although they are finally diagnosed with TM, there are multiple organ lesions, such as lymphadenopathy, anemia, leucopenia, thrombocytopenia, hepatosplenomegaly, and bone marrow involvement [16]. However, clinical case studies on TM bloodstream infection are scarce. Making an analysis about the TM bloodstream in AIDS patients will be useful for the clinicians who take care of AIDS patients. Our hospital is a referral center for AIDS patients in East China and many AIDS patients were admitted in hospital each year. The aim of this study was to retrospectively analyze the clinical characteristics of TM bloodstream infection in hospitalized AIDS patients and determine the outcomes of hospitalization after diagnosis in our hospital over the past 5 years.

## Methods

### The design and setting of the study

From January 1st 2015 to July 31th 2020, cases of TM detected by blood culture in patients admitted to the Shanghai Public Health Clinical Center (SPHCC) were sorted and analyzed. SPHCC is the designated hospital for HIV patients in Shanghai municipality and is also a tertiary referral hospital for refractory and complicated HIV-infected cases in east China. Cultures of clinical specimens were cultured on Sabouraud’s dextrose agar at 25 °C and 37 °C [17].

Electronic medical records of HIV-infected patients diagnosed with TM bloodstream infection (defined by a culture positive for TM from patient blood) were searched. Information including place of birth or long-term residence, presenting complaints and physical examinations (fever, umbilical fovea-like rash [18], and superficial lymphadenopathy), and laboratory tests (routine blood test, biochemistry, PCT, CD4 and CD8 cell counts, CRP**)** and outcomes during hospitalization were analyzed. Blood culture were performed on patients with fever, loss of weight, lymphadenopathy or other symptoms considering infection on the time of admission, and routine blood tests and tests for biochemistry, cellular immune function, PCT and CRP were measured at the same time. The patients were divided into a survival group (72 patients, getting better and alive when being discharged) and a poor prognosis group (15 patients, death or terminal discharge) according to their final clinical outcomes during the hospital admission.

### The characteristics of participants

Among 8621 cases (from January 1st 2015 to July 31th 2020) admitted to SPHCC, there were 662 positive blood culture results and including 87 cases with TM bloodstream infection.

### Statistical analysis

The rank sum test was used for the comparison of demographics and clinical characteristic data between the two groups (87 cases); chi-square analysis was used for the comparison of categorical variables. The predictors included in the multivariate model were selected based on a significance level of *p* < 0.1 in the univariate analyses (60 cases). The confounding factors retained in the multivariate model were serum PCT (> 0.60 ng/mL), CD8 count level (< 200/μl), BDG (< 100 pg/mL) and blood urea nitrogen level (> 4 mmol/L). Spearman correlation analysis was used to analyze the correlation between peripheral blood cells, albumin and the time required for TM to become positive in blood culture. The difference was statistically significant when the *P* value was < 0.05. Data analysis was conducted using IBM SPSS version 20.0 (IBM SPSS, Inc., Armonk, NY, USA).

## Results

### Case distribution and trends in TM bloodstream infection

Among 8621 cases (from January 1st 2015 to July 31th 2020) admitted to SPHCC, the rate of TM bloodstream infection among all in-hospital patients was 13 (0.99%) in 2015, and in 2019, it grew to 18 (1.01%). Since July 2020, the rate was 2.09%. Because SPHCC is a referral hospital in east China, and all TM bloodstream infection cases were scattered throughout many provinces. Zhejiang (24 cases) and Jiangxi (13 cases) were the two provinces with the largest number of TM bloodstream infection cases in this analysis.

### The comparison of clinical and laboratory features between the two groups with different clinical outcomes

The median age of patients with TM bloodstream infection was 34 years (IQR 28--44), the median hemoglobin level was 94 g/L (IQR 81--107), and the median CD4 count was 7/μl (IQR 4--19). The median time required for TM culture conversion (from taking blood culture to TM turnaround) was 8 days (IQR 6--9). The blood urea nitrogen level, PCT and CRP in the poor prognosis group were significantly higher than those in the survival group; the peripheral blood CD8 count, BDG, and serum albumin in the poor prognosis group were significantly lower than those in the survival group; the count levels of hemoglobin and CD4 in the poor prognosis group were lower than those in the survival group, but the difference was not significantly different. There was no significant difference in age or sex rate between the two groups. Among the 87 cases with TM bloodstream infection, there were 10 cases of Nontuberculous mycobacteria (NTM) disease (6 cases of pulmonary NTM disease, 3 cases of NTM blood infection, 1 case of intestinal NTM disease); 10 cases of *Pneumocystis jirovecii* pueumonia (PCP); 9 cases of oral candidiasis; 8 cases of Cytomegalovirus rentinitis (CMVR); 5 cases of tuberculosis, including 3 cases of pulmonary tuberculosis, 1 case of tuberculous lymphadenitis, and 1 case of tuberculous pleurisy; 1 case of cryptococcal meningitis. For patients with poor prognosis, the median length of hospital stay was 5 days; for survival patients, the median length of hospital stay was 22 days (See Table 1).

**Table 1:** The demographics and clinical characteristic of TM bloodstream infection AIDS patients

Demographics and clinical data	Normal range	Total cases (*n* = 87)	Survival cases (*n* = 72)	Poor prognosis (*n* = 15)	***P-***value
**Age (years)**	**–**	34.0 (28.0--44.0)	33.5 (27.3--43.0)	37.0 (30.0—46.0)	0.71
**Male (%)**	**–**	81 (93%)	68 (94%)	13 (87%)	0.28^*^
WBC (*10^9/L)	**3.5 ~ 9.5**	3.4 (2.4--5.1)	3.4 (2.4--4.9)	4.5 (2.1—8.1)	0.57
Neutrophil (*10^9/L)	**1.8 ~ 6.3**	2.9 (1.7--4.2)	2.9 (1.7--4.0)	2.4 (1.6—6.5)	0.65
**Hemoglobin (g/L)**	**115 ~ 150**	94 (81--107)	95 (81--107)	86 (80—104)	0.30
Platelet (*10^9/L)	**125 ~ 350**	100 (66--155)	99.5 (72.3--149.3)	103 (23—198)	0.60
**CD4 count (/ul)**	**410 ~ 1590**	7 (4--19) N^†^ = 84	9 (4--21) N^†^ = 71	5 (3—12) N^†^ = 13	0.05
**CD8 count (/ul)**	**190 ~ 1140**	202 (98--336) *N* = 82	223 (106--384) *N* = 70	122 (57—186) *N* = 12	0.01
**Ratio of CD4/CD8**	**0.9 ~ 3.6**	0.04 (0.02--0.10) *N* = 82	0.04 (0.02--0.10) *N* = 70	0.06 (0.03—0.10) *N* = 12	0.74
**BUN (mmol/L)**	**2.6 ~ 7.5**	4.1 (3.2--6.0) *N* = 86	4.0 (3.1--5.4) *N* = 72	6.6 (4.0—12.2) *N* = 14	0.001
**Serum Albumin (g/L)**	**40 ~ 55**	27 (23--31) *N* = 86	28 (24--31) *N* = 72	25 (22—28) *N* = 14	0.07
**PCT (ng/mL)**	**0 ~ 0.05**	0.63 (0.21--1.75) *N* = 74	0.50 (0.19--1.65) *N* = 63	1.09 (0.62—15.45) *N* = 11	0.04
**CRP (mg/L)**	**0 ~ 10**	54 (26--88) *N* = 76	44 (24--81) *N* = 63	71 (53—111) *N* = 13	0.03
**BDG (pg/mL)**	**< 60**	194 (48--264) *N* = 74	205 (101--287) *N* = 62	54 (10—222) *N* = 12	0.01
**Fever**	**–**	81 (93.1%)	67 (93.1%)	14 (93.3%)	0.97^*^
**Rash**	**–**	31 (35.6%)	26 (36.1%)	5 (33.3%)	0.84^*^
**Lymphnode enlargement**	**–**	25 (28.7%)	21 (29.2%)	4 (26.7%)	0.85^*^
**TB coinfection**	**–**	5 (5.7%)	5(6.9%)	0(0%)	0.29^*^
**NTM disease**	**–**	10(11.5%)	9 (12.5%)	1 (6.7%)	0.52^*^
**CMVR**	**–**	8 (9.2%)	8 (11.1%)	0 (0%)	0.18^*^
**PCP**	**–**	10 (11.5%)	8(11.1%)	2 (13.3%)	0.81^*^
**Cryptococcal meningitis**	**–**	1 (1.1%)	1 (1.4%)	0(0%)	0.65^*^
**Oral candidiasis**	**–**	9 (10.3%)	5 (6.9%)	4 (26.7%)	0.02^*^
**Hospital stay (days)**	**–**	21 (16–25)	22 (19–26)	5 (3–13)	< 0.0001
**Culture turnaround (days)**	**–**	8 (6--9)	8 (6--9)	6 (5--9)	0.12

* By Fisher exact test; Others by Mann-Whitney test; †N, means some of the cases with the clinical data and being collected in this analysis. ***WBC*** **white blood cell;** ***BUN*** **blood urea nitrogen;** ***PCT*** **procalcitonin;** ***CRP*** **C-reactive protein;** ***BDG*** **1,3-β-D-Glucose;** ***TB*** **tuberculosis;** ***NTM*** **Nontuberculous mycobacteria;** ***CMVR*** **Cytomegalovirus rentinitis;** ***PCP*** *Pneumocystis jirovecii* **pueumonia**

### Risk factors for TM bloodstream infection patients with poor prognosis

Of the 87 cases in the study, 27 cases with incomplete data (CD4 and CD8 cell counts, PCT, albumin) were excluded, and 60 cases left (11 with poor prognosis and 49 survival cases). The age, peripheral blood cells, serum albumin, CD4 count level, CD8 count level, and the time required for blood culture turnaround, and the PCT, BDG, and blood urea nitrogen levels of the patients were analyzed by logistic test for risk factors for poor prognosis. Univariate analysis showed that serum PCT (> 0.60 ng/mL), CD8 count level (< 200/μl), BDG (< 100 pg/mL) and blood urea nitrogen level (> 4 mmol/L) were risk factors for poor prognosis. The above four factors were included in the multivariate analysis, and the results showed that the risk of poor prognosis was 12.6 times higher in patients with CD8 count < 200/μl than in those with CD8 count > 200/μl (*p* = 0.04) and 34.9 times higher in those with BDG < 100 pg/mL than in those with BDG > 100 pg/mL (*p* = 0.01) (See Table 2).

**Table 2:** Logistic regression analysis of the risk factors for poor prognosis of patients with TM bloodstream infection

Variable	Crude odds ratio (COR)	95% confidence interval (CI)	Adjusted odds ratio (AOR)	95% confidence interval (CI)	***P***-value
**Years**	Reference	–			
≥ 35 years old	1.15	0.31--4.28			
**WBC**	Reference	–			
≥ 3.5*10^9/L	0.8	0.22--2.97			
**N**	Reference	–			
≥ 2*10^9/L	1.29	0.30--5.54			
**Hb**	Reference	–			
≥ 90 g/L	0.44	0.12--1.67			
**PLT**	Reference	–			
≥ 100*10^9/L	0.98	0.26--3.64			
**Albumin**	Reference	–			
≥ 26 g/L	0.28	0.07--1.09			
**CD4**	Reference	–			
≥ 5/ul	0.44	0.12--1.67			
**CRP**	Reference	–			
≥ 65 mg/L	3.01	0.77--11.73			
**Culture conversion (days)**	Reference	–			
≥ 6 days	0.24	0.04--1.27			
**PCT**	Reference	–	Reference	–	–
≥ 0.60 ng/mL	5.09	1.00--26.00	8.47	0.55--129.41	0.13
**CD8**	Reference	–	Reference	–	–
< 200/ul	6.53	1.27--33.46	12.64	1.08--148.54	0.04
**BDG**	Reference	–	Reference	–	–
< 100 pg/mL	4.68	1.18--18.51	34.85	2.34--519.10	0.01
**BUN**	Reference	–	Reference	–	–
≥ 4 mmol/L	5.52	1.08--28.25	2.4	0.30--19.00	0.41

***WBC*** **white blood cell;** ***N*** **neutrophil;** ***Hb*** **hemoglobin;** ***PLT*** **platelet;** ***PCT*** **procalcitonin;** ***CRP*** **C-reactive protein;** ***BDG*** **1,3-β-D-Glucose;** ***BUN*** **blood urea nitrogen**

### The correlation between laboratory tests and the time of TM turnaround in blood culture

Spearman correlation analysis was performed with the time to TM positive conversion in blood culture as the dependent variable and the levels of peripheral leukocytes, neutrophils, hemoglobin, platelets, albumin and PCT, and BDG as independent variables. The results showed that the levels of both platelets (r = 0.37, *p* = 0.0004) and albumin (r = 0.44, *p* < 0.0001) were positively correlated with the time required for TM blood culture turnaround, and the levels of PCT (r = − 0.40, *p* = 0.0009) and BDG (r = − 0.30, *p* = 0.0076) were negatively correlated with the time required for TM positive in blood culture.

## Discussion

The rate of TM bloodstream infection among admitted patients increased from 1.0% in 2015 to 2.1% in 2020 (from January to July) at SPHCC. According to a systematic review by Qin et al. [13], the estimated pooled prevalence of TM infection in China was 3.3% (95% CI: 1.8–5.8%), and the prevalence in Shanghai was 1.8% (95% CI: 1.3–2.4%), which are in line with our findings. As a referral center, we treat many kinds of opportunistic infections, including TM, and based on the etiology study of bloodstream infection among in-hospital patients in 2016 [3], TM bloodstream infection accounted for 18.8% (43/299), which was the second most common bloodstream infection pathogen. With the progress of AIDS treatment and physician awareness of TM, reports of AIDS with TM infection have become increasingly common, and the prognosis depends on a timely diagnosis [19] and proper antifungal treatment [20, 21]. In this study, the mortality rate of patients with TM bloodstream infection was 17.2% (15/87), which is similar to 17.5% (191/1093) published by Jiang et al. in 2019 [6] and 16.7% [17] in our hospital from 2014 to 2015. In view of the fact that this is a single-center study from a tertiary hospital [22] where patients from all parts of the country obtain further diagnosis and treatment, it cannot represent all the patients with TM disease.

In this study, the level of blood urea nitrogen, PCT, and CRP in the poor prognosis group were significantly higher than those in the survival group; the peripheral blood CD8 count level in the poor prognosis group were significantly lower than those in the survival group; in the overall presentation of patients with TM bloodstream infection, although they have immunodeficiency, poor prognosis patients are often in an extremely severe immunodeficient state and have poor nutritional status compared with the survival patients. Among the 15 cases of worsening illness or death in this study, the median length of hospital stay was only 5 days, which means that at the time of terminal discharge or death, TM disease often had not been confirmed, which also indicates that the immunodeficiency caused by HIV infection requires early diagnosis and early intervention to avoid the development of severe immunodeficiency [23].

The presence of TM bloodstream infection is an indicator of severe immunodeficiency [4, 24]. When they are in such an immunodeficient state, patients often have coinfections, such as cytomegalovirus retinitis, pulmonary TB, NTM disease, and cryptococcal meningitis [22, 25]. There were fewer cases of coinfection in the group with poor prognosis. Considering that the group with poor prognosis had shorter overall hospitalization time and worsened or even died soon after admission, resulting in insufficient time for diagnosis, there may be missed diagnoses and fewer confirmed diseases than those in the group of surviving patients. Patients with poor prognosis are mostly in the late stage of the disease. Although they were given supportive treatment after hospitalization, their condition still got worse, and they lost the chance for antifungal treatment (or other comorbidities fail to control, and their condition is incurable). Therefore, the hospitalization days for patients with poor prognosis was significantly shorter than that of normal discharged patients.

In this study, risk factor analysis of patients with poor prognosis after clinical treatment for TM bloodstream infection showed that patients with CD8 count < 200/μl and whole blood BDG < 100 pg/mL had 12.6 times and 34.9 times the risk of poor prognosis, respectively. The median CD4 count was only 7/μl, which belongs to the extremely immunodeficient population; the CD8 cell count was still significantly reduced, and the median count was only 202/μl. CD8 counts may predict prognosis independently of CD4 counts [26]. In most cases, the end stage of HIV infection would cause both CD4 and CD8 depletion [27]. The delay in the diagnosis of TM independently predicted the early mortality of these patients [28–30].

This study found that those with a BDG less than 100 pg/mL are at risk for poor prognosis. As mentioned previously, the BDG assay is helpful in the early detection of invasive fugal disease [31, 32], including TM [33]. However, the elevation of BDG also have been linked to gut damage with increased intestinal permeability in HIV patients [34, 35]. Furthermore, we found BDG was negatively correlated with the time required for blood culture turnaround in our analysis. This unexpected findings may be explained by the small sample size and need further investigation.

There are some limitations of this study. First, because this is a retrospective analysis, the clinical data, such as treatment regimen administered before admission in our hospital, records of patients’ history of travel to an endemic area, as well as the time from onset of symptoms to diagnosis, all affect the accuracy of descriptions and analysis. Second, this was a single-center study, and caution should be paid to extrapolating study findings to the whole population in areas with a high incidence of TM disease in China. Thirdly, the outcome would be affected by other opportunistic infections, however, these factors were not retained in the multivariate model because of limited cases with coinfections.

## Conclusions

Bloodstream infections caused by TM are becoming increasingly common in the AIDS population with severe immunodeficiency in East China. For those with extremely low CD4 and CD8 cell counts below 200/ul are with an increased risk of poor prognosis. Improving the awareness of symptoms such as umbilical fovea rash, fever and lymphadenopathy has a positive effect on early diagnosis of the disease and optimization of treatment efficacy.

## Data Availability

The datasets used and/or analyzed during the current study are available from the corresponding author on reasonable request.

## Abbreviations

TM: Talaromyces marneffei
HIV: Human immunodeficiency virus
AIDS: Acquired immunodeficiency syndrome
WBC: White blood cell
BUN: Blood urea nitrogen
PCT: Procalcitonin
CRP: C-reactive protein
BDG: 1,3-β-D-Glucose
TB: Tuberculosis
NTM: Nontuberculous mycobacteria
CMVR: Cytomegalovirus rentinitis
PCP: *Pneumocystis jirovecii* pueumonia
